# CT Coronary Angiography Is Feasible for the Assessment of Coronary Artery Disease in Chronic Dialysis Patients, Despite High Average Calcium Scores

**DOI:** 10.1371/journal.pone.0067936

**Published:** 2013-07-10

**Authors:** Mihály K. de Bie, Maurits S. Buiten, André Gaasbeek, Mark J. Boogers, Cornelis J. Roos, Joanne D. Schuijf, M. Jacqueline Krol, Ton J. Rabelink, Jeroen J. Bax, Martin J. Schalij, J. Wouter Jukema

**Affiliations:** 1 Department of Cardiology, Leiden University Medical Center, Leiden, The Netherlands; 2 Department of Nephrology, Leiden University Medical Center, Leiden, The Netherlands; 3 Department of Nephrology, Haga Hospital, den Haag, The Netherlands; University of Washington School of Medicine, United States of America

## Abstract

**Purpose:**

Significant obstructive coronary artery disease (CAD) is common in asymptomatic dialysis patients. Identifying these high risk patients is warranted and may improve the prognosis of this vulnerable patient group. Routine catheterization of incident dialysis patients has been proposed, but is considered too invasive. CT-angiography may therefore be more appropriate. However, extensive coronary calcification, often present in this patient group, might hamper adequate lumen evaluation. The objective of this study was to assess the feasibility of CT-angiography in this patient group.

**Methods:**

For this analysis all patients currently participating in the ICD2 trial (ISRCTN20479861), with no history of PCI or CABG were included. The major epicardial vessels were evaluated on a segment basis (segment 1–3, 5–8, 11 and 13) by a team consisting of an interventional and an imaging specialist. Segments were scored as not significant, significant and not interpretable.

**Results:**

A total of 70 dialysis patients, with a mean age of 66±8 yrs and predominantly male (70%) were included. The median calcium score was 623 [79, 1619].

Over 90% of the analyzed segments were considered interpretable. The incidence of significant CAD on CT was 43% and was associated with cardiovascular events during follow-up. The incidence of cardiovascular events after 2-years follow-up: 36% vs. 0% in patients with no significant CAD (p<0.01).

**Conclusion:**

Despite the high calcium scores CT-angiography is feasible for the evaluation of the extent of CAD in dialysis patients. Moreover the presence of significant CAD on CT was associated with events during follow-up.

## Introduction

Survival of patients on dialysis treatment is abysmal. [1] Coronary artery disease (CAD) probably plays an important role in this poor survival and is highly prevalent among dialysis patients.[2]–[4] Noteworthy, the currently reported prevalence is even an underestimation, given the high prevalence of CAD among asymptomatic dialysis patients. Indeed, in several studies high prevalences of CAD of ∼40% to 50% have been observed, even in asymptomatic dialysis patients.[5]–[7] Furthermore, it was reported that the coronary artery lesions are often (>65% of the cases) located in the proximal parts of the epicardial vessels, which is strongly associated with diminished survival. [6], [8] Identification of these high risk patients is therefore warranted and might improve outcome. Given the high cardiovascular mortality and high prevalence of CAD in asymptomatic patients, routine catheterization of new dialysis patients has been proposed. [5] However, catheterization is associated with significant risks and costs and therefore less invasive diagnostic strategies would probably be more appropriate for these asymptomatic patients. CT angiography (CTA) of the coronary arteries may be such an alternative. In non dialysis patients CTA has proven to be feasible and to have good specificity and even better sensitivity for determining the presence of CAD.[9]–[11] However, in dialysis patients, data regarding the potential value of CTA are lacking. Furthermore, there are concerns that CTA may be less feasible given the extent of vessel calcification in this patient group.[12]–[14] On the other hand, recent data indicates that even in patients with severe coronary calcification, sensitivity and specificity of CTA remain high. [15] Moreover, in dialysis patients, vascular calcification occurs not only in the intima of the vessel wall, but also in the media of the vessel wall. The consequences of these two forms of calcification differ: intima calcification leads to vascular occlusion whereas calcification of the media leads to vascular stiffening, but does not affect luminal narrowing. [16], [17] It is therefore conceivable that calcifications may have less effect on the feasibility of CTA in dialysis patients than is currently supposed. The objective of this study was to evaluate the feasibility of CTA to assess the severity of CAD, in the proximal segments of the coronary arteries, in this vulnerable patient population.

## Methods

### Study Population

For this analysis all patients enrolled in the ICD2 trial (ISRCTN20479861) between may 2007 and October 2011, who were referred for CT angiography, were included. The rationale and methods of this study have previously been reported. [18] In short, this study is designed to evaluate the effectiveness of prophylactic ICD implantation for the prevention of sudden cardiac death in dialysis patients. Patients enrolled in this study undergo an extensive screening protocol at baseline including CT angiography, transthoracic echocardiography and vascular function assessment. Events during follow-up are recorded and graded by an independent clinical event committee. Patients with previous coronary artery bypass grafts or percutaneous coronary interventions with stents were excluded from the current analysis, since the goal of this study is to identify unknown CAD. Patients with atrial fibrillation, or patients with a heart rate above 80 bpm after administration of oral β-blockers, were also not considered for this analysis. The ICD2 study protocol has been approved by the local ethics committee (Commissie Medische Ethiek, Leids Universitair Medisch Centrum) and all participating patients provided written and oral consent.

### Multi Slice CT Protocol

Examinations were performed with a 64-detector row CT Scanner (Aquilion 64, Toshiba Medical Systems, Tokyo, Japan) or a 320-detector row CT scanner (Aquilion ONE, Toshiba, Tokyo, Japan). In patients with a heart rate >65 bpm oral β-blockers (metroprolol 50 or 100 mg, single dose, 1 hour before examination) were administered, if tolerated. If tolerated, patients were also administered a single dose of nitroglycerin.

A non-enhanced low-dose electrocardiographically gated scan was performed, prior to the helical scan, to measure coronary calcium score (CCS). The CCS scan was prospectively triggered at 70% or 75% of the R-R interval. For the 64-row CT the scan was performed using the following scan parameters: 4×3.0 mm or 2.5 mm collimation for 64-row CT, and single rotation wide volume acquisition (320×0.5 mm, reconstructed to 3 mm slices). For the 320-row CT: gantry rotation time, 350–500 ms; tube voltage, 120 kV; and tube current, 200–250 mA.

CTA examinations were performed as follows. On the Aquilion 64 CT-coronary angiograpy was performed after an injection of 90–100 ml non-ionic contrast (Iomeron 400; Bracco, Milan, Italy), via the antecubital vein, at a flow rate of 4–6 mL/sec, which was followed by a bolus chaser of 50 mL of saline at the same flow rate. A bolus-tracking technique was used to determine the initiation of the CT data acquisition. The protocol consisted of the use of the following: collimation 64×0.5 mm; gantry rotation time 400–500 ms; tube voltage 120/135 kV; tube current 250–400 mA.

All images were acquired during a single inspiratory breath hold of 10 seconds, while the electrocardiogram was registered simultaneously. Based on a segmental reconstruction algorithm, data of one, two or three consecutive heartbeats were used to generate a single image. Images were reconstructed most often in the end-diastolic phase, since this is typically the phase showing the least motion artifacts. However, additional reconstructions were made throughout the entire cardiac cycle, when needed.

Examinations on the Aquilion ONE were performed as follows: A triphasic intravenous injection of 60–80 mL a nonionic contrast medium (Iomeron 400; Bracco, Milan, Italy) was administered in the antecubital vein. Initially, 50 to 70 mL (depending on body weight) of the contrast medium was administered at a flow rate of 5.0 or 6.0 mL/s. This was followed by 20 mL of 50% contrast/saline mix. Finally, a saline flush of 25 mL was administered at a flow rate of 3.0 mL/s.

The protocol consisted of the use of the following: collimation 320×0.5 mm; gantry rotation time 350 ms; voltage 100–135 kV; a tube current of 400–580 mA.

In order to reduce radiation dose, data were acquired using prospective triggering. In patients with a stable heart rate <60 bpm the phase window was set at 75% of R-R interval, in patients with a heart rate between 60 and 65 bpm the phase window was set to 65% and 85%. In patients with a heart rate >65 bpm CT angiography acquisition was performed during multiple heart beats with a phase window of 30% to 80% of the R-R interval. Images were reconstructed at 75% of the R-R interval. If acquisition was performed at a wider R-R interval, additional reconstructions were explored in case of motion artifacts, to obtain images with the least motion artifacts.

Depending on the patients residual kidney function the following measures were taken in order to prevent further kidney function deterioration: adequate pre and post procedural hydration (dose and route depending on the patients residual kidney function) and in hemodialysis patients the scan was performed on the day prior to the next dialysis session.

### MSCT Data Analysis

The coronary calcium score was analyzed using the Agatston method. CTA image analysis was performed by 2 experienced CT observers, an imaging specialist and an intervention cardiologist. If there was no consensus between them a 3^rd^ independent reviewer was consulted. Datasets were evaluated visually on the axial slices, assisted by 3D volume rendered reconstructions and curved multiplanar reconstructions. For this study all major epicardial segments were analyzed: in the RCA segments 1–3; in the LAD segments 5–8; and in the LCx segments 11 and 13. First the interpretability of each segment was assessed. Calcified artery segments were considered interpretable as long as a reasonable interpretation of the lumen could be accomplished. If this was not possible, mainly due to blooming artifacts of the calcium, the segment was considered uninterpretable. As with all segments, if there was no consensus regarding the interpretability between the 2 reviewers a third independent reviewer was consulted.

**Figure 1:**
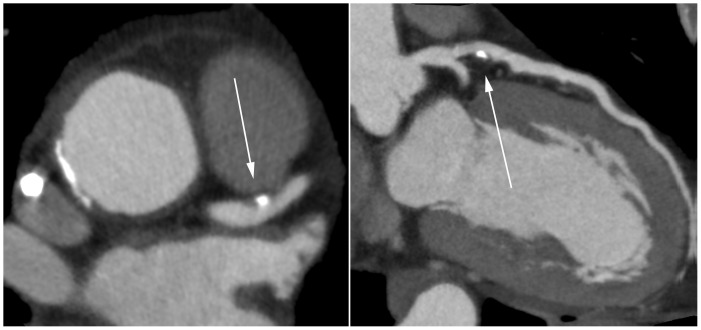
Non significant lesion of the proximal LAD. (Left panel: axial reconstruction; Right panel curved multiplanar reconstruction.).

**Figure 2:**
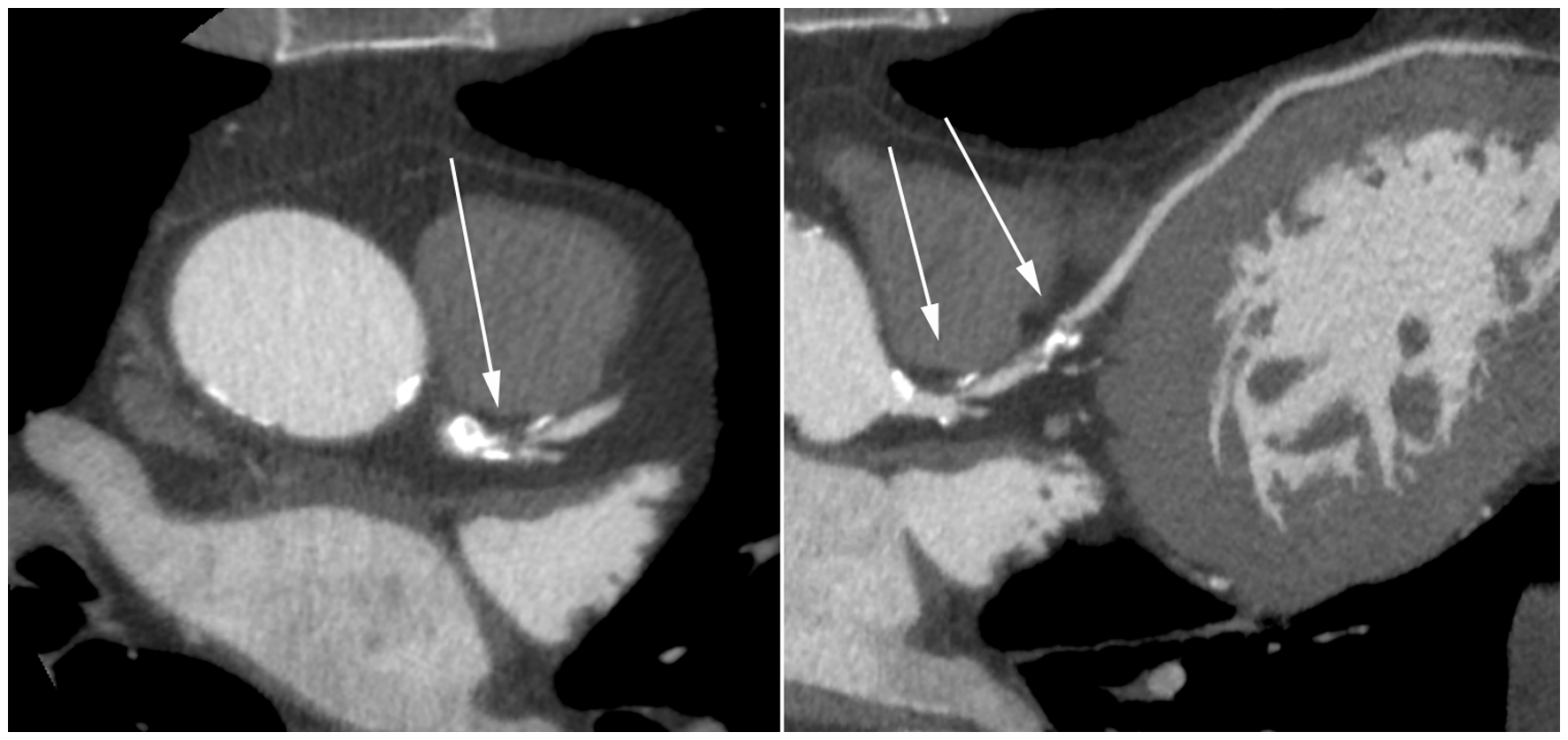
Pin-point mixed lesion of the left main and significant lesion of the proximal LAD. (Left panel: axial reconstruction; Right panel curved multiplanar reconstruction.).

**Figure 3:**
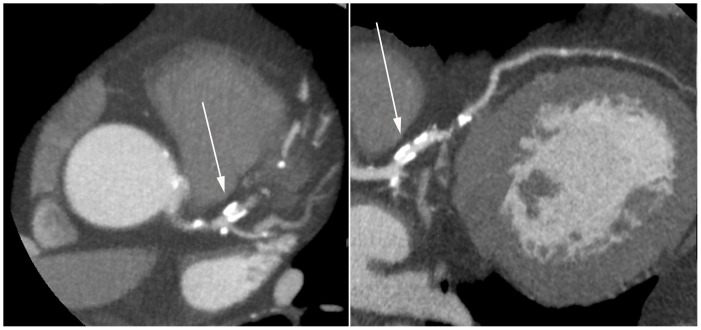
Non-interpretable lesion of the proximal LAD. (Left panel: axial reconstruction; Right panel curved multiplanar reconstruction.).

When considered interpretable, the degree of luminal narrowing was determined and graded as not significant (<50% luminal narrowing) or significant (≥50%). If more than one segment in a vessel was considered non-interpretable, the vessel was considered non-interpretable. If more than one vessel was considered non-interpretable, the entire scan was considered non-interpretable. Figure 1 and 2 show an example of interpretable segments without and with significant CAD. Figure 3 is an example of a non-interpretable segment.

### Follow-up and End Points

Events were closely monitored and judged by an independent clinical event committee. For this study we used the combined endpoint fatal myocardial infarction, non-fatal myocardial infarction and revascularization. (Non-)fatal infarction was defined based on criteria of typical chest pain, elevated cardiac enzyme levels and typical changes on the ECG. [19].

### Statistics

Data are presented as mean ± SD. All variables were normally distributed (as assessed by the Kolgmorov-Smirnov test), except coronary artery calcium score and dialysis vintage. Continous data were compared using the 2-tailed Student’s t-test (for normally distributed variables) for paired data or the Mann-Whitney U test (for non-normally distributed variables). Categorical data were compared using the Chi-square test. All statistical analyses were performed Using SPSS (version 18.0, SPPS Inc. Chicago, Illinois). Cumulative event rates were assessed using the method of Kaplan-Meier and compared using the log rank test. All statistical tests were two-sided and p-values <0.05 were considered statistically significant. All statistical analyses have been performed in PASW statistics version 18.0.

## Results

For this study, out of the 108 patients currently enrolled in the ICD2 study, a total 70 patients were included. Twenty patients were excluded because of history of CABG, 9 patients because of history of PCI, 6 patients because of a high/irregular heart rate and 3 patients refused CTA. The main clinical characteristics of the patients included in this analysis are summarized in table 1. Patients were predominantly male (49 patients, 70%), with a mean age of 66±8 years. The median coronary artery calcium score (CACS) was 623 [79, 1619].

**Table 1:** Patient Characteristics.

**Age, yrs.**	66±8
**Male gender, %(nr)**	70% (49)
**Patients on heamodialysis, % (nr)**	64% (45)
**Patients on peritoneal dialysis, % (nr)**	36% (25)
**Dialysis vintage, months**	16 [9, 29]
**BMI (kg/m^2^)**	26.6±4.5
**Current Smoker, %**	26% (18)
**Diabetes Mellitus, % (nr)**	24% (17)
**Hypertension, % (nr)**	74% (52)
**β-blocker, % (nr)**	46% (32)
**ACEi/AT2i,% (nr)**	61% (43)
**Calcium antagonist, % (nr)**	44% (31)
**Statin, % (nr)**	56.0% (39)
**CT - Calcium Score**	623 [79, 1619]

### CT – Results

In total 627 segments were analyzed of which 573 (91.4%) were considered interpretable.

Nineteen of the 54 non-interpretable segments were considered non-interpretable because of extensive calcification. The remaining 35 segments were considered non-interpretable due to technical reasons, including motion artifacts and poor contrast arrival. On a per vessel basis, when defined as ≤1 non-interpretable segment, 195 (92.8%) vessels were considered interpretable. At least 2 vessels were considered interpretable in 67 (95.7%) patients, and subsequently these scans were considered interpretable.

A significant lesion was found in 96 (15.3%) segments and in 30 (42.9%) patients at least one significant lesion was present. Table 2 summarizes the per segment outcome of the scans.

**Table 2:** Per segment analysis outcome.

*Segment*	<50%	≥50%	Non - interpretable
**1**	55 (78.6%)	12 (17.1%)	3 (4.3%)
**2**	48 (69.6%)	11 (15.9%)	10 (14.5%)
**3**	55 (80.9%)	10 (14.7%)	3 (4.4%)
**5**	66 (94.3%)	2 (2.8%)	2 (2.8%)
**6**	45 (64.3%)	18 (25.7%)	7 (10.0%)
**7**	46 (65.7%)	18 (25.7%)	6 (8.6%)
**8**	54 (77.1%)	8 (11.4%)	8 (11.4%)
**11**	56 (80.0%)	9 (12.9%)	5 (7.1%)
**13**	52 (74.3%)	8 (11.4%)	10 (14.3%)
**Total**	**477 (76.1%)**	**96 (15.3%)**	**54 (8.6%)**

### Follow-up

The average follow-up after acquisition of the CT-scan was 22±14 months. During this follow-up period 6 patients reached the composite end-point of (non)-fatal myocardial infarction and revascularization. One patient died due to acute myocardial infarction, 2 patients underwent PCI after being admitted to the hospital with acute coronary syndrome, 2 patients underwent elective PCI because of the transplantation work-up and 1 patient underwent elective CABG in combination with aortic valve replacement. In all 6 patients significant CAD was documented with CT angiography and confirmed with coronary angiography prior to, or at time of the event. No events occurred in patients in whom no significant CAD was documented with CT angiography.

**Figure 4:**
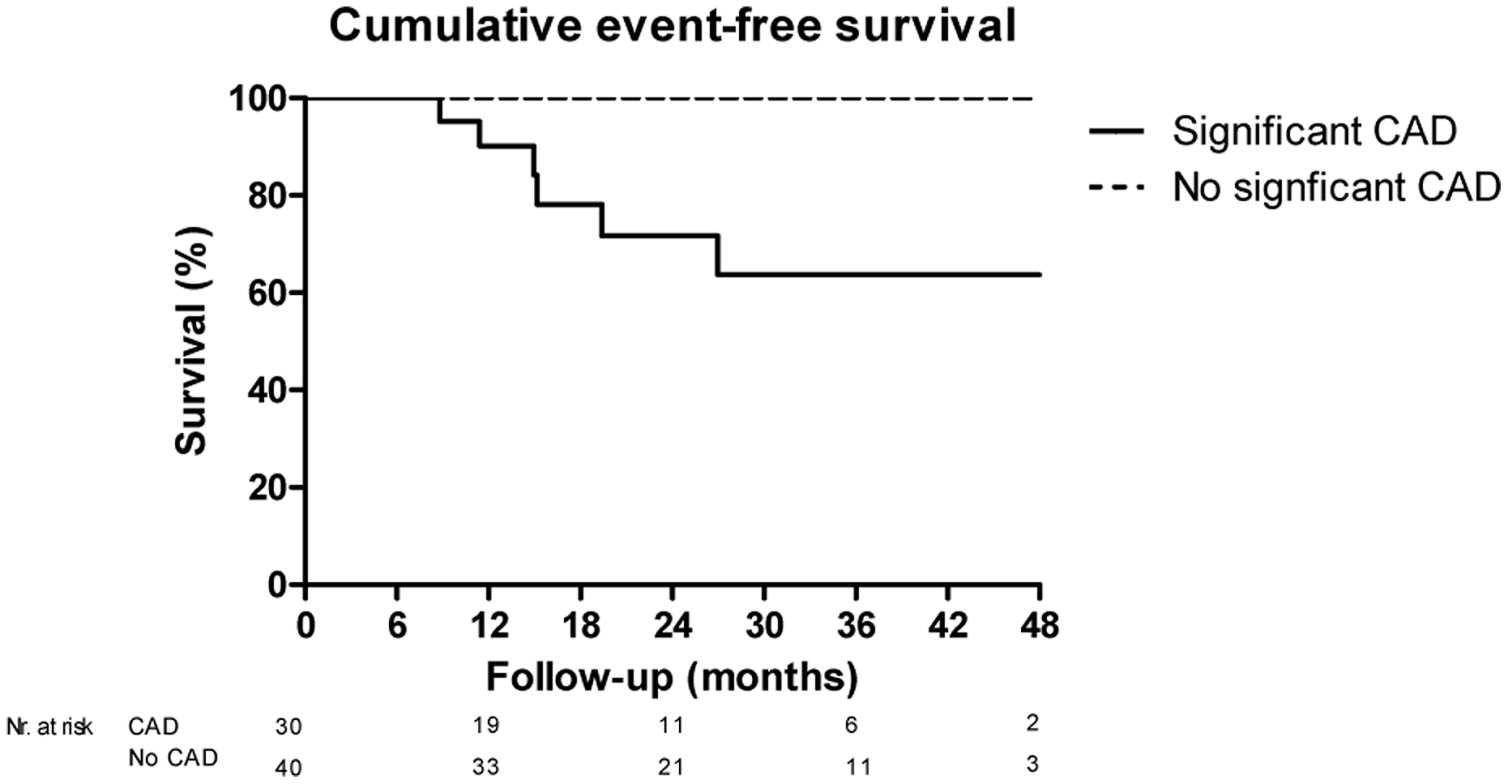
Kaplan Meier cure for the cumulative event rate of the primary endpoint in patients with significant CAD on CT vs. no significant CAD on CT.

There was a significant difference in the incidence of the primary endpoint. The Kaplan Meier estimated incidence of cardiovascular events after 2-years follow-up was 36% (95%CI 12%–60%) in patients with documented significant CAD on CT compared to no events in patients with no significant CAD on CT (p<0.01). Figure 4.

### Predictors for Non-interpretable Segments

In total 19 patients had ≥1 segment that was considered non-interpretable. Patients with non-interpretable segment(s) had a significant higher BMI (29.8 vs. 25.4 kg/m^2^, p<0.001) compared to patients without non-interpretable segments. There were no other significant differences between patients with and without non-interpretable segments. In particular there were no significant differences with regard to heart rate during scanning and CACS. Table 3.

**Table 3:** Differences in patients with and without non-interpretable segments.

	All segmentsInterpretable(N = 51)	≥1 Non-interpretableSegment. (N = 19)	P value
**Age, yrs.**	67±8	66±8	p = 0.79
**Male gender, %(nr)**	71% (36)	68% (13)	p = 0.86
**Haemodialysis, % (nr)**	59% (30)	79% (15)	p = 0.12
**Peritoneal dialysis, % (nr)**	41% (21)	21% (4)	p = 0.12
**Dialysis vintage, months**	19 [9, 29]	12 [8, 32]	p = 0.46*
**Heart rate during scan (bpm)** **	60±8	63±8	p = 0.19
**BMI (kg/m^2^)**	25.4±3.4	29.8±5.4	p<0.001
**Diabetes Mellitus, % (nr)**	22% (11)	32% (6)	p = 0.39
**Hypertension, % (nr)**	73% (37)	79% (15)	p = 0.59
**CT - Calcium Score**	594 [49, 1618]	636 [225, 1714]	P = 0.39*

* Mann Whitney U-test;

** Not available for 2 patients.

### Safety of CT

None of the patients experienced anaphylactic reactions which required intervention. Furthermore in patients with residual kidney function no sudden decrease in residual function (decrease of 24/h urine production) that could be related to the CT-procedure was observed.

## Discussion

The key finding of this study is that CT angiography, of the proximal parts of the coronary arteries, seems well feasible for the assessment of CAD, for the majority of dialysis patients. Moreover, significant CAD at CT-angiography was associated with events during follow-up. Furthermore, this study confirms the high incidence of significant CAD in asymptomatic dialysis patients.

### Screening Asymptomatic Dialysis Patients for the Presence of Significant CAD

Screening for disease in asymptomatic patient groups can only be defended when there is a significant prevalence of the disease among asymptomatic patients and furthermore, an intervention should be available that could improve outcome when applied during the asymptomatic phase. As mentioned, the prevalence of significant CAD among asymptomatic dialysis patients is approximately 40–50%, indicating, that based on the prevalence of the disease screening seems warranted.[5]–[7] Whether intervention, either by PCI or by CABG, improves outcome in asymptomatic dialysis patients with proven CAD is not entirely sorted out. However, there are several reports that indicate that intervention with PCI or CABG for proven CAD drastically improves outcome in chronic dialysis patients. [20], [21].

### Selecting the Optimal Screening Strategy

Coronary angiography is the current gold standard for the diagnosis of CAD. However, coronary angiography has several drawbacks that make it less preferable for screening in asymptomatic patients. For instance coronary angiography is associated with high costs and is an invasive procedure with concomitant risk of complications. These complications include stroke, arrhythmias, local complications at the puncture site, atheroembolism and contrast induced acute kidney injury. Given the risks and costs another less invasive screening tool would probably be more appropriate. Several other tests are available to detect the presence of significant CAD in dialysis patients, however, each of them has their own specific drawbacks. For instance exercise tolerance testing is often not feasible in dialysis patients because the target heart rate can not be achieved and other tests such as dobutamine stress echocardiography and myocardial perfusion scintigraphy have relatively low sensitivity, especially in dialysis patients. [22].

### Multi Slice Computer Tomography

Multi Slice Computed Tomography has proven to have good diagnostic properties in non-dialysis patients with especially high sensitivity, making it an ideal tool to rule out significant CAD. [10] However, it is known that heavily calcified segments, often present in dialysis patients might give false positive results. [12], [13] On the other hand in contrast to patients with normal renal function, in whom calcification occurs in the intima of the vessel, vascular calcification in dialysis patients is often also related to the media of the vessel. [16], [17] Since this type of calcification does not influence luminal narrowing, the luminal evaluation in dialysis might remain feasible despite the severe (media) calcification that is often seen in dialysis patients. Furthermore, as was recently demonstrated in a meta-analysis investigating the sensitivity and specificity of novel CT systems, sensitivity and specificity remain high in case of severe coronary calcification. One of the factors potentially influencing interpretability is the distribution of coronary calcification. The authors of the meta-analysis suggest if a high calcium score is formed because of diffuse calcifications than this is less likely to result to result in non-interpretability compared to when considerable calcification is limited to a small area. [15] Since in dialysis patients vascular calcification is a generalized problem this might also (partially) explain the finding that CT-calcium score did not significantly differ between patients in whom all segments were considered interpretable and those in whom 1 or more segments were considered non-interpretable.

In this study evaluation of the presence of CAD was considered feasible in ∼90% of the analyzed segments. The presence of significant CAD found on CT was associated with future cardiovascular events, whereas in patients in whom no significant CAD was found, no cardiovascular events occurred.

### Study Limitations

Since only very limited data on CTA in dialysis patients is currently available, [23] a study that assesses the diagnostic accuracy of CTA, before strengthening the hypothesis that CTA indeed might be of clinical value, was in our opinion not ethical. Therefore the purpose of this study was first to assess whether CTA gives interpretable results and furthermore whether these findings relate to clinical end-points. The diagnostic accuracy of CTA compared to coronary angiography was thus not yet assessed. This drawback limits conclusions about the impact of the severity of CAD documented per segment. Nevertheless, since no events occurred in patients in whom no significant CAD was detected with CAD, we feel that CTA might be an appropriate tool to rule out the presence of severe CAD in dialysis patients. In order to define the true clinical value of CTA more studies regarding the diagnostic accuracy of CTA in dialysis patients are warranted.

### Clinical Implications

The potential of CTA lies in the fact that it is a very useful tool to rule out significant coronary artery disease. Although high calcium scores may result in more false positives, the sensitivity for detecting significant CAD of CTA in heavily calcified segments seems high. Although it cannot be concluded based on the current results, CTA in dialysis patients (with severely calcified coronary arteries) probably will result in a number of false positive results. However bearing in mind that it is suggested that coronary catheterization should be performed in all asymptomatic dialysis patients, we feel that CTA nonetheless optimizes patient selection for coronary angiography and that patients in whom significant CAD is demonstrated are good candidates for catheterization.

### Conclusion

Despite the severe coronary calcification in dialysis patients, CTA seems feasible for the assessment of CAD, as over 90% of the analyzed segments were considered interpretable. Furthermore the presence of CAD on CT was associated with a 2-year cumulative incidence of cardiovascular events of approximately 30% whereas patients with no significant CAD experienced no cardiovascular events. Finally, the high prevalence of significant CAD in asymptomatic dialysis patients was confirmed with the current analysis.

## References

[1] U.S. Renal Data System, USRDS 2006 Annual Data Report: Atlas of Chronic Kidney Disease and End-Stage Renal Disease in the United States, National Institutes of Health, National Insitute of Diabetes and Digestive and Kidney Diseases, Bethesda, MD.

[2] Rocco MV, Yan G, Gassman J, Lewis JB, Ornt D, et al (2002) Comparison of causes of death using HEMO Study and HCFA end-stage renal disease death notification classification systems. The National Institutes of Health-funded Hemodialysis. Health Care Financing Administration. Am J Kidney Dis 39: 146–153. 11774113 10.1053/ajkd.2002.29905

[3] Cheung AK, Sarnak MJ, Yan G, Berkoben M, Heyka R, et al (2004) Cardiac diseases in maintenance hemodialysis patients: results of the HEMO Study. Kidney Int 65: 2380–2389. 15149351 10.1111/j.1523-1755.2004.00657.x

[4] Foley RN, Parfrey PS, Sarnak MJ (1998) Clinical epidemiology of cardiovascular disease in chronic renal disease. Am J Kidney Dis 32: S112–S119. 9820470 10.1053/ajkd.1998.v32.pm9820470

[5] Joki N, Hase H, Nakamura R, Yamaguchi T (1997) Onset of coronary artery disease prior to initiation of haemodialysis in patients with end-stage renal disease. Nephrol Dial Transplant 12: 718–723. 9141000 10.1093/ndt/12.4.718

[6] Charytan D, Kuntz RE, Mauri L, DeFilippi C (2007) Distribution of coronary artery disease and relation to mortality in asymptomatic hemodialysis patients. Am J Kidney Dis 49: 409–416. 17336702 10.1053/j.ajkd.2006.11.042

[7] Ohtake T, Kobayashi S, Moriya H, Negishi K, Okamoto K, et al (2005) High prevalence of occult coronary artery stenosis in patients with chronic kidney disease at the initiation of renal replacement therapy: an angiographic examination. J Am Soc Nephrol 16: 1141–1148. 15743997 10.1681/ASN.2004090765

[8] Joki N, Hase H, Takahashi Y, Ishikawa H, Nakamura R, et al (2003) Angiographical severity of coronary atherosclerosis predicts death in the first year of hemodialysis. Int Urol Nephrol 35: 289–297. 15072511 10.1023/b:urol.0000020356.82724.37

[9] Carrigan TP, Nair D, Schoenhagen P, Curtin RJ, Popovic ZB, et al (2009) Prognostic utility of 64-slice computed tomography in patients with suspected but no documented coronary artery disease. Eur Heart J 30: 362–371. 19153177 10.1093/eurheartj/ehn605

[10] Mowatt G, Cook JA, Hillis GS, Walker S, Fraser C, et al (2008) 64-Slice computed tomography angiography in the diagnosis and assessment of coronary artery disease: systematic review and meta-analysis. Heart 94: 1386–1393. 18669550 10.1136/hrt.2008.145292

[11] de Graaf FR, Schuijf JD, van Velzen JE, Kroft LJ, de RA, et al (2010) Diagnostic accuracy of 320-row multidetector computed tomography coronary angiography in the non-invasive evaluation of significant coronary artery disease. Eur Heart J 31: 1908–1915. 20047991 10.1093/eurheartj/ehp571

[12] Ghostine S, Caussin C, Daoud B, Habis M, Perrier E, et al (2006) Non-invasive detection of coronary artery disease in patients with left bundle branch block using 64-slice computed tomography. J Am Coll Cardiol 48: 1929–1934. 17112979 10.1016/j.jacc.2006.04.103

[13] Raff GL, Gallagher MJ, O’Neill WW, Goldstein JA (2005) Diagnostic accuracy of noninvasive coronary angiography using 64-slice spiral computed tomography. J Am Coll Cardiol 46: 552–557. 16053973 10.1016/j.jacc.2005.05.056

[14] Braun J, Oldendorf M, Moshage W, Heidler R, Zeitler E, et al (1996) Electron beam computed tomography in the evaluation of cardiac calcification in chronic dialysis patients. Am J Kidney Dis 27: 394–401. 8604709 10.1016/s0272-6386(96)90363-7

[15] den Dekker MA, de SK, de Bock GH, Tio RA, Oudkerk M, et al (2012) Diagnostic performance of coronary CT angiography for stenosis detection according to calcium score: systematic review and meta-analysis. Eur.Radiol. 22: 2688–2698. 10.1007/s00330-012-2551-x 22797978

[16] Cannata-Andia JB, Rodriguez-Garcia M, Carrillo-Lopez N, Naves-Diaz M, Diaz-Lopez B (2006) Vascular calcifications: pathogenesis, management, and impact on clinical outcomes. J Am Soc Nephrol 17: S267–S273. 17130273 10.1681/ASN.2006080925

[17] Goldsmith D, Ritz E, Covic A (2004) Vascular calcification: a stiff challenge for the nephrologist: does preventing bone disease cause arterial disease? Kidney Int. 66: 1315–1333. 10.1111/j.1523-1755.2004.00895.x 15458425

[18] de Bie MK, Lekkerkerker JC, van Dam B, Gaasbeek A, van Buren M, et al (2008) Prevention of sudden cardiac death: rationale and design of the Implantable Cardioverter Defibrillators in Dialysis patients (ICD2) Trial–a prospective pilot study. Curr Med Res Opin 24: 2151–2157. 18561878 10.1185/03007990802237343

[19] Thygesen K, Alpert JS, White HD, Jaffe AS, Apple FS, et al (2007) Universal definition of myocardial infarction. Circulation 116: 2634–2653. 17951284 10.1161/CIRCULATIONAHA.107.187397

[20] Hemmelgarn BR, Southern D, Culleton BF, Mitchell LB, Knudtson ML, et al (2004) Survival after coronary revascularization among patients with kidney disease. Circulation 110: 1890–1895. 15451786 10.1161/01.CIR.0000143629.55725.D9

[21] Yasuda K, Kasuga H, Aoyama T, Takahashi H, Toriyama T, et al (2006) Comparison of percutaneous coronary intervention with medication in the treatment of coronary artery disease in hemodialysis patients. J Am Soc Nephrol 17: 2322–2332. 16837633 10.1681/ASN.2005090958

[22] De Vriese AS, Vandecasteele SJ, Van den Bergh B, De Geeter FW (2011) Should we screen for coronary artery disease in asymptomatic chronic dialysis patients? Kidney Int. 10.1038/ki.2011.340 21956188

[23] Iio K, Nagasawa Y, Kimura T, Yamasaki K, Takeda Y, et al (2008) Assessment of coronary stenosis by a 16-slice MDCT scanner in asymptomatic diabetic patients starting dialysis therapy. Nephron Clin Pract 109: c72–c79. 18560241 10.1159/000139992

